# Successful Engraftment Following Blinatumomab and Donor-Derived Buffy Coat Infusion in a Patient With Relapsed B-cell Acute Lymphoblastic Leukemia and High-Level Donor-Specific Anti-HLA Antibodies: A Case Report

**DOI:** 10.7759/cureus.115374

**Published:** 2026-08-28

**Authors:** Takahiro Maeta, Kazuya Asano, Kentaro Kobayashi, Tsuyoshi Sato, Shigeki Ito

**Affiliations:** 1 Hematology and Oncology, Iwate Medical University, Yahaba, JPN

**Keywords:** b-cell acute lymphoblastic leukemia, blinatumomab, buffy coat infusion, donor-specific anti-hla antibodies, graft failure, haploidentical hematopoietic stem cell transplantation

## Abstract

A 40-year-old Japanese woman with Philadelphia chromosome-negative B-cell acute lymphoblastic leukemia (B-ALL) underwent human leukocyte antigen (HLA)-matched related peripheral blood stem cell transplantation approximately 22 months before the current transplantation because measurable residual disease persisted after induction therapy. She experienced hematologic relapse approximately six months before the current transplantation and achieved complete remission after two cycles of inotuzumab ozogamicin. A second transplantation using her HLA-haploidentical son as the donor was planned; however, pretransplant testing detected donor-specific anti-HLA antibodies (DSA) against HLA-A31 and HLA-B51, with mean fluorescence intensity (MFI) values of 10,582 and 19,288, respectively. Five doses of rituximab reduced the MFI values for HLA-A31 and HLA-B51 to 2,341 and 9,789, respectively. Two cycles of blinatumomab produced a further reduction, although the HLA-B51 MFI remained above 3,000. An irradiated donor-derived buffy coat prepared from 400 mL of whole blood was therefore infused one day before stem cell infusion, followed by haploidentical hematopoietic stem cell transplantation. Neutrophil engraftment was achieved on day 9, complete donor chimerism was confirmed on day 28, and DSA became undetectable on day 33. No secondary graft failure or severe acute graft-versus-host disease occurred. This case suggests that blinatumomab followed by donor buffy coat infusion may be considered as part of a multimodal desensitization strategy in selected patients with B-ALL and persistent clinically significant DSA. However, the efficacy and safety of this approach cannot be established from a single case.

## Introduction

Allogeneic hematopoietic stem cell transplantation (HSCT) is a potentially curative treatment for hematologic malignancies, but graft failure is a life-threatening complication. Pre-existing donor-specific anti-human leukocyte antigen (HLA) antibodies (DSAs) are associated with an increased risk of graft failure, particularly after cord blood transplantation and HLA-haploidentical transplantation [1-3]. DSAs are thought to impair engraftment through antibody-mediated injury to donor hematopoietic stem and progenitor cells and potentially other donor-derived or stromal elements that support hematopoiesis, with complement activation and antibody-dependent cellular cytotoxicity contributing to graft rejection [1]. Accordingly, HLA antibody screening is important when considering an HLA-mismatched bone marrow or peripheral blood stem cell graft.

The clinical significance of a DSA depends not only on its presence but also on its strength, persistence, and functional characteristics, such as complement-binding activity. Although DSA strength is commonly assessed using mean fluorescence intensity (MFI) in solid-phase single-antigen bead assays, MFI is a semiquantitative, assay-dependent measure rather than a direct measure of antibody concentration. Its interpretation may be influenced by factors such as antigen density, assay saturation, and the prozone effect; therefore, no single MFI threshold uniformly defines clinically significant DSA across transplantation settings. Nevertheless, high-level and persistent DSAs, particularly those with complement-binding capacity, are generally considered to confer a greater risk of graft failure [1-3].

Reported desensitization strategies include rituximab, plasma exchange, high-dose intravenous immunoglobulin, bortezomib, and antibody neutralization using donor HLA antigens, such as donor-compatible platelet or cellular-product transfusion [4]. The effectiveness of these approaches is variable, and their availability may differ across healthcare systems. A previous Japanese case report described a marked reduction in broad anti-HLA antibodies after blinatumomab-containing therapy in a patient with B-cell acute lymphoblastic leukemia (B-ALL), permitting subsequent allogeneic HSCT [5]. Blinatumomab is a bispecific T-cell engager that redirects CD3-positive T-cells toward CD19-positive B-lineage cells and is effective in relapsed or refractory B-ALL [6]. However, evidence regarding its effect on pre-existing HLA antibodies remains limited.

Here, we report a patient with relapsed B-ALL who had persistent high-level DSAs against the available haploidentical donor. Although an unrelated donor search was performed through the donor registry, no suitable alternative donor was identified. Thus, the patient faced a difficult clinical situation in which transplantation with the haploidentical donor carried a substantial risk of graft failure because of persistent DSAs, while switching to a DSA-negative donor was not feasible. We therefore used a sequential desensitization strategy consisting of rituximab and blinatumomab therapy, followed by a donor-derived buffy coat infusion, before haploidentical HSCT. The patient subsequently achieved successful donor engraftment and was followed until day 607 after transplantation. This case highlights the potential utility of combining B-cell-directed therapy with donor-antigen-based antibody neutralization in patients with clinically significant DSAs when no suitable alternative donor is available.

## Case presentation

A 40-year-old Japanese woman was diagnosed with Philadelphia chromosome-negative B-ALL at Iwate Medical University Hospital, Iwate, Japan, approximately 29 months before the current transplantation. She received induction therapy with dexamethasone, vincristine, doxorubicin, cyclophosphamide, L-asparaginase, and prednisolone according to the Japan Adult Leukemia Study Group ALL202-O protocol [7]. Because measurable residual disease remained detectable after induction, she underwent HLA-matched related peripheral blood stem cell transplantation from her older brother approximately 22 months before the current transplantation. Complete donor chimerism was confirmed on day 28, and she subsequently maintained complete remission until hematologic relapse approximately 16 months after the first transplantation.

In preparation for a second allogeneic HSCT, an unrelated donor search was performed through the donor registry; however, no suitable donor was identified. Therefore, her son, a 16-year-old male haploidentical related donor, was selected. The donor and recipient were mismatched at 5 of 8 HLA loci.

HLA antibody testing was performed using the LABScreen assay (One Lambda), which detects IgG antibodies against HLA antigens and assesses panel-reactive antibody (%PRA). HLA antibody specificities were further determined separately for HLA class I and class II using single-antigen bead assays. According to the laboratory criteria, beads with a normalized MFI ≥1,000 were considered positive. The patient's HLA antibody specificities were compared with the prospective donor's HLA typing results to perform a virtual crossmatch and identify DSAs. Complement-binding DSA testing, such as C1q- or C3d-binding assays, was not performed.

The patient was readmitted for salvage therapy with inotuzumab ozogamicin [8]. Her vital signs and physical examination findings were unremarkable. The white blood cell count was 20,560/μL, with 35.0% blasts. The serum lactate dehydrogenase level was elevated, consistent with active leukemia (Table 1) [9]. Electrocardiography, chest radiography, and noncontrast computed tomography revealed no clinically significant abnormalities.

**Table 1:** Laboratory findings on admission at the time of relapse after transplantation

Investigation	Result	Reference range
White blood cell count	20,560/μL	3,300-8,600 /μL
Neutrophil	29.00%	49.7-72.7%
Lymphocyte	34.50%	24.5-38.9%
Monocyte	1.00%	1.7-8.7%
Eosinophil	0.50%	0.0-5.0%
Blast	35.00%	-
Red blood cell count	342 × 10^4 ^/μL	386-492 × 10^4 ^/μL
Hemoglobin	10.9 g/dL	11.6-14.8 g/dL
Mean corpuscular volume	96.4 fL	83.6-98.2 fL
Platelet count	5.1 × 10^4^ /μL	15.8-34.8 × 10^4^ /μL
Reticulocyte	1.1‰	3.6-22.0‰
Albumin	4.2 g/dL	4.1-5.1 g/dL
Sodium	143 mmol/L	138-145 mmol/L
Potassium	4.0 mmol/L	3.6-4.8 mmol/L
Chloride	109 mmol/L	101-108 mmol/L
Calcium	9.0 mg/dL	8.8-10.1 mg/dL
Blood urea nitrogen	14.1 mg/dL	8-20 mg/dL
Creatinine	0.65 mg/dL	0.49-0.79 mg/dL
Aspartate aminotransferase	27 U/L	13-30 U/L
Alanine aminotransferase	38 U/L	7-23 U/L
Lactate dehydrogenase	296 U/L	124-222 U/L
Gamma-glutamyl transferase	55 U/L	9-32 U/L
Cholinesterase	264 U/L	201-421 U/L
Alkaline phosphatase	79 U/L	106-322 U/L
Total bilirubin	0.2 mg/dL	0.4-1.5 mg/dL
C-reactive protein	0.48 mg/dL	<0.14 mg/dL

The baseline MFI values of the DSAs against HLA-A31 and HLA-B51 were 10,582 and 19,288, respectively. After two cycles of inotuzumab ozogamicin, complete remission was achieved. One dose of rituximab (375 mg/m²/day) was then administered for desensitization, after which the HLA-A31 and HLA-B51 MFI values decreased to 4,038 and 11,594, respectively.

Blinatumomab was subsequently administered as bridging therapy before transplantation. Because cytokine release syndrome developed, blinatumomab was maintained at 9 μg/day throughout the first 28-day cycle. Two doses of rituximab were administered; nevertheless, the HLA-A31 and HLA-B51 MFI values remained 2,341 and 9,789, respectively. After two cycles of blinatumomab and two additional doses of rituximab, a further decline was observed, although the HLA-B51 MFI remained above 3,000.

No suitable unrelated bone marrow or cord blood donor was available; therefore, the patient proceeded to transplantation using her HLA-haploidentical son as the donor. Plasma exchange was considered an additional desensitization strategy. However, at the time of treatment, pretransplant DSA desensitization was not routinely reimbursed under the Japanese national health insurance system. It was not available as part of routine clinical care at our institution. Because clinically significant DSA against HLA-B51 persisted, an irradiated donor-derived buffy coat was administered 1 day before stem cell infusion as an additional desensitization measure (Figure 1). The patient received a conditioning regimen consisting of fludarabine at 25 mg/m²/day from day −7 to day −3, melphalan at 70 mg/m²/day on days −3 and −2, and total body irradiation at 3 Gy on day −1. Graft-versus-host disease prophylaxis consisted of antithymocyte globulin, tacrolimus, and methylprednisolone. The buffy coat was prepared by centrifugation of 386 mL of donor whole blood, yielding a final volume of 162 mL. The product was ABO-mismatched to the recipient. The total white blood cell count was 1.03 × 10⁶ per bag. The buffy coat was infused 24 hours before stem cell infusion, and no infusion-related adverse events were observed.

**Figure 1:**
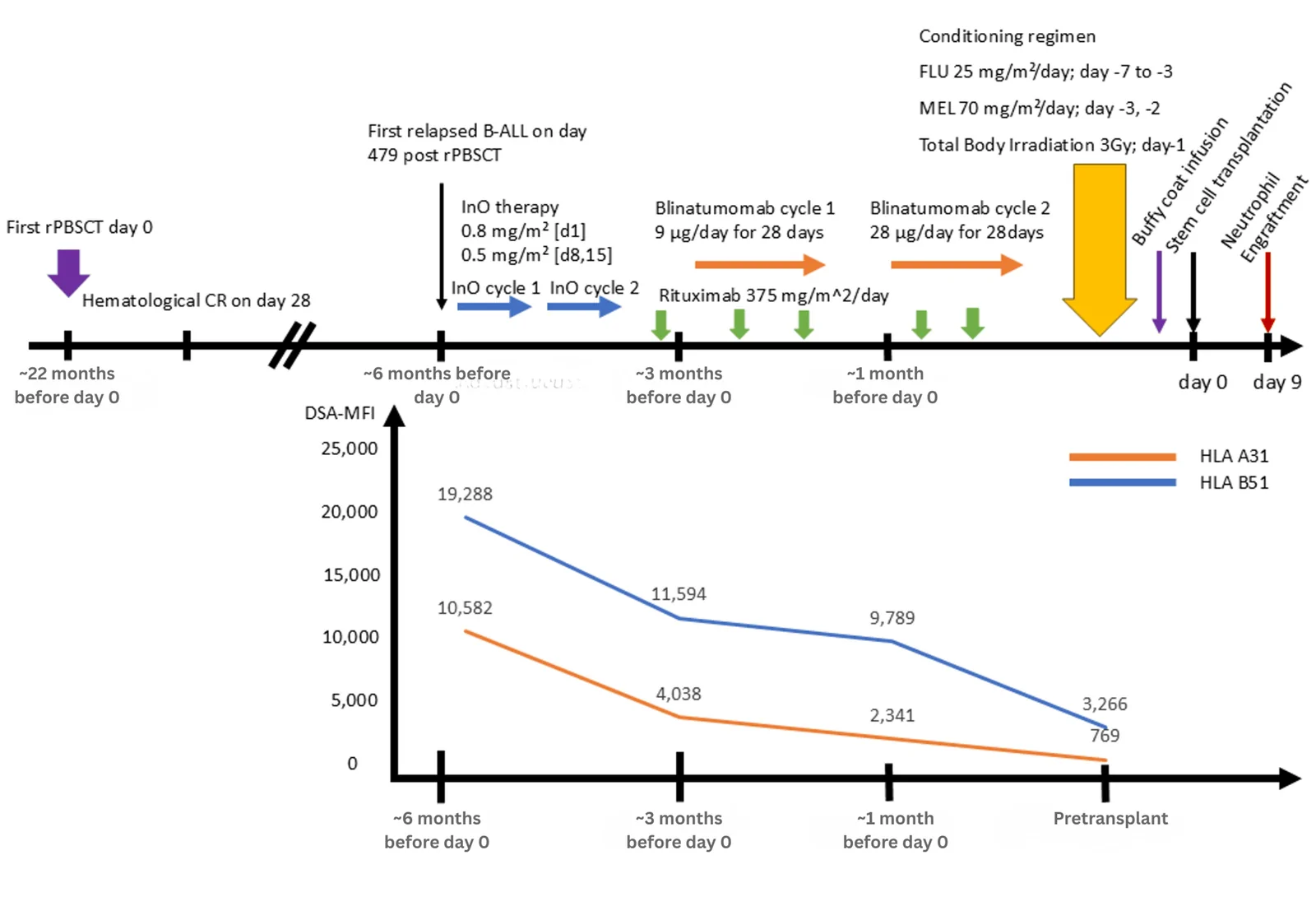
Clinical course and trends in DSA MFI PBSCT: peripheral blood stem cell transplantation, CR: complete remission, B-ALL: B-cell acute lymphoblastic leukemia, InO: inotuzumab ozogamicin, DSA: donor-specific anti-HLA antibody, MFI: mean fluorescence intensity, HLA: human leukocyte antigen, FLU: fludarabine, MEL: melphalan This figure was created using PowerPoint 365 and Excel 365 (Microsoft Corporation, Redmond, WA, USA).

The patient underwent haploidentical HSCT on day 0 using a graft from a 16-year-old male donor. The infused CD34-positive cell dose was 4.57 × 10^6 cells/kg. HLA typing of the patient revealed HLA-A 02:01/02:06, HLA-B 40:02/48:01, HLA-C 03:04/08:03, and homozygosity for HLA-DRB1 04:05. The donor HLA type was HLA-A 02:06/31:01, HLA-B 48:01/51:01, HLA-C 08:03/14:02, and HLA-DRB1 04:05/08:02. The patient was positive for cytomegalovirus (CMV) IgG antibodies. In contrast, the donor was negative for CMV IgG antibodies. The recipient and donor had blood groups B Rh-positive and O Rh-positive, respectively, indicating a minor ABO mismatch. Neutrophil engraftment was achieved on day 9 after transplantation. Bone marrow examination on day 28 confirmed complete remission and complete donor chimerism. On day 33, the MFI values of both DSAs were below the assay detection limit. At the latest follow-up, on day 607 after the second transplantation, the patient remained in complete remission with complete donor chimerism and no evidence of secondary graft failure.

## Discussion

This case describes successful haploidentical HSCT in a patient with relapsed B-ALL and persistent high-level DSAs after a sequential desensitization strategy consisting of rituximab, blinatumomab, and donor-derived buffy coat infusion. Although repeated rituximab administration partially reduced the DSA levels, the HLA-B51 MFI remained above 9,000. Blinatumomab produced a further reduction, but the HLA-B51 MFI remained above 3,000 before transplantation. Buffy coat infusion was therefore added immediately before stem cell infusion. Neutrophil engraftment occurred on day 9, complete donor chimerism was confirmed on day 28, and DSA was undetectable on day 33.

Pre-existing DSAs are associated with reduced engraftment after cord blood and haploidentical transplantation [1,2,10,11]. In one study of haploidentical transplantation, an anti-HLA antibody MFI of 10,000 or higher was associated with an increased risk of graft failure [2]. However, the DSA level that permits safe transplantation remains uncertain. MFI is semiquantitative, varies among assay platforms and laboratories, and should be interpreted in the context of antibody specificity, strength, complement-binding capacity when available, and the clinical transplantation setting [2,4]. Patients without an alternative donor may need to proceed to transplantation despite persistent DSA.

Several desensitization approaches have been reported, including rituximab-based regimens, donor-derived cellular products, plasma exchange, intravenous immunoglobulin, and proteasome inhibition [4,12,13]. Rituximab depletes CD20-positive B-cells, including memory B-cells, but has limited direct activity against plasmablasts and plasma cells that express little or no CD20 [14]. This biologic limitation may explain why DSA persisted despite repeated rituximab administration in the present case.

In contrast, CD19 is expressed across much of the B-cell lineage and may be retained by some plasmablasts and short-lived antibody-secreting cells [15,16]. Blinatumomab links CD19-positive B-lineage cells to CD3-positive T-cells, resulting in T-cell-mediated cytotoxicity [17]. Reductions in circulating B-cells and immunoglobulin levels have been reported during CD19-directed therapy [18,19]. Together with the serial MFI changes in our patient, these observations suggest that blinatumomab may suppress HLA antibody production by depleting or functionally suppressing CD19-positive B-lineage populations. Nevertheless, the available evidence is limited to small observational reports, and blinatumomab should not be regarded as a stand-alone desensitization strategy for all patients with clinically significant DSA.

Donor-derived buffy coat infusion may provide a substantial amount of donor HLA antigen that binds circulating DSA, thereby promoting antibody neutralization or clearance [4]. In the present case, this intervention was used because DSA persisted despite rituximab and blinatumomab and no suitable alternative donor was available. Prompt engraftment and disappearance of DSA after transplantation are clinically encouraging; however, the contribution of the buffy coat cannot be separated from the effects of the preceding therapies, the conditioning regimen, and the donor graft.

This report has several limitations. It describes a single patient who received multiple sequential interventions, making it impossible to determine the relative contribution of each treatment to the reduction in DSA levels or subsequent successful engraftment. Therefore, the observed outcomes cannot be specifically attributed to blinatumomab or to donor-derived buffy coat infusion, and no causal relationship can be established from this single case. MFI values are semiquantitative, and complement-binding DSA testing was not performed. Additionally, the follow-up period presented here is limited. Additional case reports/series and prospective evaluations are needed to determine the optimal candidates, timing, dosing, safety, and DSA targets for this strategy.

## Conclusions

In this case, a patient with relapsed B-ALL had persistent high-level DSAs against the only available haploidentical donor, creating a substantial barrier to transplantation. Sequential rituximab and blinatumomab therapy followed by donor-derived buffy coat infusion was associated with reductions in DSA levels and successful engraftment after haploidentical HSCT. The decline in DSA after blinatumomab suggests that CD19-directed therapy may contribute to desensitization, while buffy coat infusion may provide an additional donor-specific antibody-neutralizing effect. However, because this was a single case involving multiple sequential interventions, the contribution of each component cannot be determined, and the efficacy and safety of this approach cannot be established. Nevertheless, this case highlights the potential utility of combining B-cell-directed therapy with donor-antigen-based antibody neutralization as a multimodal desensitization strategy for carefully selected patients with clinically significant DSA when no suitable alternative donor is available.
